# Multi-stage surgery to treat oesophageal fistula that developed after chemoradiotherapy in two patients with oesophageal cancer: A case report

**DOI:** 10.1016/j.ijscr.2024.109460

**Published:** 2024-03-04

**Authors:** Tomoaki Furuta, Katsuji Hisakura, Koichi Ogawa, Yoshimasa Akashi, Jaejeong Kim, Tatsuya Oda

**Affiliations:** a Department of Gastrointestinal and Hepatobiliary–Pancreatic Surgery, Faculty of Medicine, University of Tsukuba, Tsukuba 305-8575, Japan; b Department of Surgery, Hitachi Ltd., Hitachinaka General Hospital, Hitachinaka, Ibaraki 312-0057, Japan

**Keywords:** Oesophageal fistulae, Oesophageal cancer, Chemoradiotherapy, Multi-stage surgery, Case repor

## Abstract

**Introduction and importance:**

Oesophageal fistula is a severe complication that may occasionally develop after chemoradiotherapy (CRT) for oesophageal cancer and is difficult to treat.

**Case presentation:**

A 51-year-old man who had undergone CRT for oesophageal cancer presented with haematemesis and was diagnosed with a descending aortic aneurysm and an oesophageal fistula. Thoracic endovascular aneurysm repair was performed to achieve haemostasis. After 3 days, the patient underwent subtotal oesophagectomy and cervical oesophagostomy with delivery of a pedicled omental flap to the exposed aortic stent. Six months later, ileocecal reconstruction was performed. The second patient was a 49-year-old woman who had undergone CRT 1 year previously. She complained of leg weakness and gait disorder. After a workup, she was diagnosed with perforation of the posterior wall of the cervical oesophagus with abscess formation and purulent spondylitis. After two spinal fusion surgeries, we performed tracheotomy and drained the cervical region to reduce local infection. After 7 days, she underwent pharyngolaryngoesophagectomy and reconstruction using a gastric conduit, to which a large section of the omental flap was attached. After the multi-stage surgery, oral intake became possible, and both patients were discharged.

**Clinical discussion:**

The optimal treatment strategy for post-CRT oesophageal fistula remains controversial. Radical surgery, including oesophagectomy, is the treatment of choice, although it is associated with high mortality rates. Multi-stage surgery may be useful for reducing surgical stress in moribund patients.

**Conclusion:**

We reported two cases involving radiation-induced oesophageal fistula successfully treated by multi-stage surgery without major complications.

## Highlights

• Multi-stage surgery can successfully treat radiation-induced oesophageal fistula.
• Multi-stage surgery allows for infection control.
• Multi-stage surgery reduces surgical stress in moribund patients.
• Two cases of radiation-induced oesophageal fistula were successfully treated.

## 1 Introduction

Chemoradiotherapy (CRT) is a standard treatment for oesophageal cancer; however, its use is limited because it causes several adverse reactions. Oesophageal fistula develops following therapeutic intervention for oesophageal cancer in approximately 6 %–22 % cases, and its prognosis is very poor [1,2]. Per previous studies, the mean survival time is only 2–2.5 months [3,4]. Stent placement is often performed for the treatment of oesophageal fistula, although certain complications such as massive haemorrhage and infection have been reported. Current treatment strategies for oesophageal fistulae are ineffective [5], and to our knowledge, no studies have reported the usefulness of surgery for the treatment of CRT-induced oesophageal fistula in patients with oesophageal cancer.

Here we report two cases involving middle-aged patients who developed oesophageal fistula after CRT for oesophageal cancer and were successfully treated with multi-stage surgery.

## 2 Case presentation

### 2.1 Case 1

**Fig. 1:**
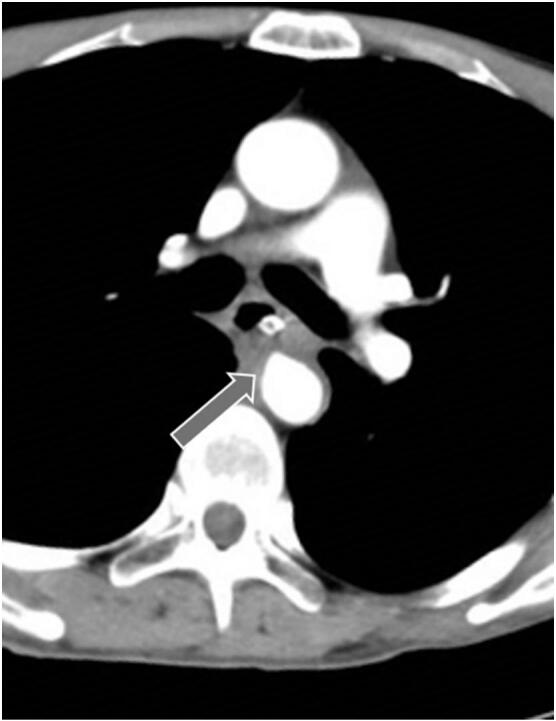
Case 1: a 51-year-old man with oesophageal fistula that developed after chemoradiotherapy for oesophageal cancer. Computed tomography performed on arrival reveals an aneurysm in the descending aorta (arrow) adjacent to the thoracic oesophagus.

**Fig. 2:**
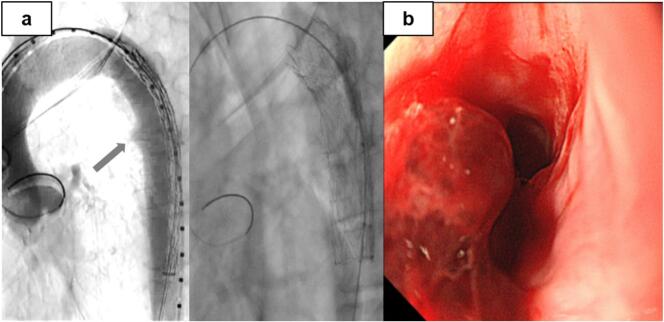
Case 1: a 51-year-old man with oesophageal fistula that developed after chemoradiotherapy for oesophageal cancer. a, Thoracic endovascular aneurysm repair is performed to achieve complete haemostasis for treating the aneurysm in the descending aorta (arrow). B, Haemostasis is confirmed by endoscopy after removal of the Sengstaken–Blakemore tube.

**Fig. 3:**
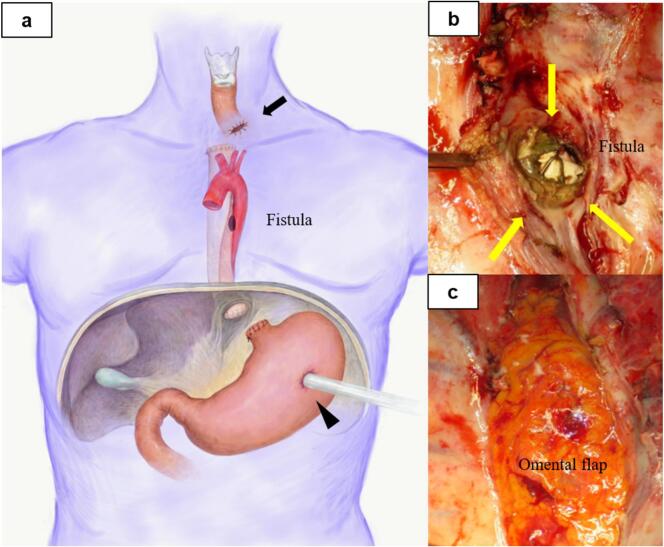
Case 1: a 51-year-old man with oesophageal fistula that developed after chemoradiotherapy for oesophageal cancer. a, Transthoracic resection of the thoracic oesophagus is followed by cervical oesophagostomy (arrow) and tube gastrostomy (arrow head). b, The aortic stent is exposed through the fistula in the field of oesophageal resection (yellow arrow). c, A pedicled omental flap delivered through the abdomen is patched onto the aortic stent exposed through the fistula. (For interpretation of the references to colour in this figure legend, the reader is referred to the web version of this article.)

A 51-year-old man was admitted to a nearby hospital with massive haematemesis and haemorrhagic shock. He had a history of middle thoracic oesophageal cancer (squamous cell carcinoma, cT4N0M0) and had undergone CRT involving a total dose of 60-GyE proton beams administered in 30 fractions administered with cisplatin (CDDP) and 5-fluorouracil (5-FU). Approximately 4 months after CRT completion, he was urgently transported to the hospital with massive haematemesis. He was diagnosed with haemorrhagic shock and immediately treated with a Sengstaken–Blakemore (S–B) tube for temporary haemostasis. Computed tomography (CT) revealed a descending aortic aneurysm with an oesophageal fistula (Fig. 1). After primary haemostasis and continuous blood transfusion, he was transferred to our hospital for subsequent treatment. On arrival, his haemoglobin level was 5.1 mg/dL, haematocrit was 15.4 %, platelet count was 14.4 × 10^4^/μL (>50 % decrease from counts recorded at the previous hospital), and prothrombin time was 30.9 % (international normalized ratio, 2.02), consistent with a diagnosis of disseminated intravascular coagulation (DIC). Thoracic endovascular aneurysm repair (TEVAR) was performed urgently to achieve complete haemostasis (Fig. 2A), which was confirmed by endoscopy after S–B tube removal (Fig. 2B). On day 4, following improvements in shock and DIC, we performed subtotal oesophagectomy, cervical oesophagostomy, and tube gastrostomy (Fig. 3A) with delivery of a pedicled omental flap to the aortic stent exposed through the fistula (Fig. 3B, C). Histopathological examination showed that in the penetrated area, the epithelium was replaced by fibrous connective tissue with inflammatory cell infiltration. No residual carcinoma was observed in the resected oesophagus, which was expected after radiation-induced injury. Aortic replacement was considered, but the patient showed no increase in the inflammatory response and no relapse of inflammation after antimicrobial therapy for 1 month. Therefore, ileocecal reconstruction was performed without complications 3 months later. Seven days after reconstructive surgery, imaging using oral contrast showed no abnormalities, and oral intake was possible. The patient was discharged without tube feeding 15 days after reconstructive surgery. Five years after reconstruction, the patient showed good disease control without any readmission related to oesophageal cancer treatment.

### 2.2 Case 2

**Fig. 4:**
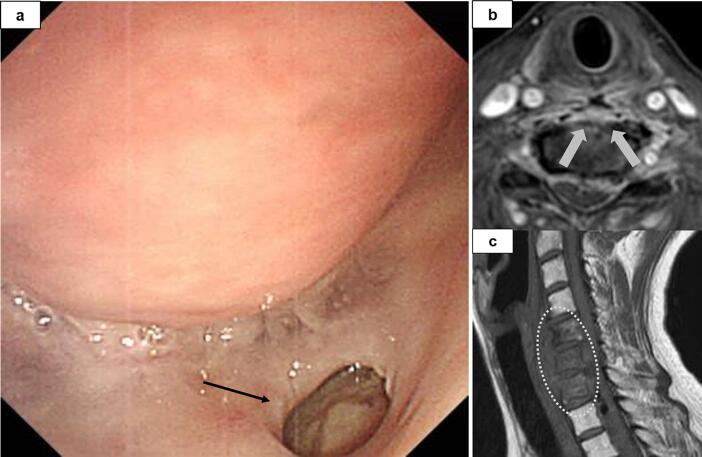
Case 2: a 49-year-old woman with oesophageal fistula that developed after chemoradiotherapy for oesophageal cancer. a, Endoscopic examination reveals a perforation in the posterior wall of the cervical oesophagus (black arrow). b, Magnetic resonance imaging shows the perforation, with an abscess cavity (arrow). c, The C5-7 vertebral body is involved in the cervical abscess (surrounding dotted-line) and destroyed.

**Fig. 5:**
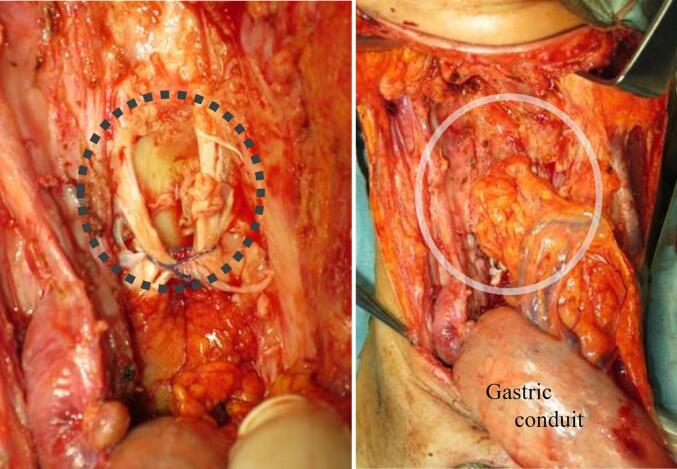
Case 2: a 49-year-old woman with oesophageal fistula that developed after chemoradiotherapy for oesophageal cancer. Pharyngolaryngoesophagectomy reveals the destroyed vertebral body, which was previously repaired by spinal fusion surgery (surrounding dotted line) and patched using an omental flap delivered to the cervical lesion through the gastric conduit (surrounding white circle).

A 49-year-old woman with a history of cervical oesophageal cancer (squamous cell carcinoma, cT2N0M0) who had undergone CRT with a total dose of 30-GyE X-rays and 30-GyE proton beams with CDDP and 5-FU 1 year previously visited our hospital with neck pain, leg weakness, and gait disorder. Urgent endoscopy and magnetic resonance imaging (MRI) revealed perforation of the posterior wall of the cervical oesophagus with abscess formation and purulent spondylitis (Fig. 4). Blood tests showed a white blood cell count of 17,900 cells/μL and C-reactive protein level of 17.4 mg/dL. Broad-spectrum antibiotic therapy was initiated, and on days 0 and 2 after admission, she underwent posterior decompression and spinal fusion surgery, which were performed on a semi-urgent basis for purulent spondylitis. On day 7, tracheotomy and cervical lesion drainage were performed because the general inflammation did not improve after surgery. On day 14, her white blood cell count and C-reactive protein level were 8900 cells/μL and 2.5 mg/dL, respectively. Following considerable reduction in the general inflammation, she underwent pharyngolaryngoesophagectomy and reconstruction using a gastric conduit, to which a large section of the omental flap was attached. The omental flap was delivered to the cervical lesion through the gastric conduit and patched onto the damaged vertebra (Fig. 5). Histopathologically, fibrin was seen adhered to the surface of the torn tissue at the perforated site, with evidence of acute inflammation mainly including neutrophils in the surrounding area. The tissue damage occurred from the adventitial side of the oesophagus, and no recurrence was observed in the resected oesophagus. The patient started tube feeding on day 2 after reconstruction, and no abnormality was observed on imaging with oral contrast on day 8; thus, oral intake was resumed. Tube feeding was not required after 4 weeks, and the feeding tube was removed at 6 weeks. Following reconstruction, Clavien–Dindo grade II (CD-II) urinary infection, upper gastrointestinal haemorrhage, and atrial fibrillation occurred; however, they were quickly controlled. The patient was transferred to a rehabilitation institution 70 days after reconstruction. Three years after reconstruction, she showed good disease control without any oesophageal cancer-related readmissions.

The retrospective protocol was approved by our institutional review board (No. R1-017), and written informed consent for publication was obtained from the patients.

## 3 Discussion

Oesophageal fistula to adjacent organs, such as the aorta, vertebra, trachea, and pericardium, are rarely caused by radiotherapy; however, it is associated with high mortality rates. Radiotherapy damages the walls of the oesophagus and adjacent organs through delayed injury and may induce fistula formation [6]. In this report, we present two cases of oesophageal fistulae caused by CRT that were successfully treated with multi-stage surgery using an omental flap.

In terms of long-term effectiveness and durability, radical surgery, including oesophagectomy, is generally the treatment of choice for oesophageal fistula because conventional therapy is ineffective [7]. However, oesophagectomy is invasive and continues to be associated with high mortality rates [8]. In contrast, multi-stage surgeries reportedly reduce surgical stress by reducing blood loss and operation time, given the division of a major invasive procedure into multiple gradual surgeries [9]. Two-stage surgery for oesophageal cancer seems beneficial for patients at risk of impaired vascular gastric perfusion or those with borderline functional operability due to single or multiple organ dysfunctions [10]. Recently, Yoshimura et al. reported that orthopaedic surgery followed by two-stage oesophagectomy was successfully performed to treat oesophageal perforation after CRT for non-small cell lung cancer with a mediastinal abscess penetrating the vertebral disc [11]. Two-stage surgery for oesophageal fistula is recommended to eliminate the risk of septic shock because emergency intervention in debilitated patients is a risk factor for anastomotic leakage, that can lead to septic shock [12]. In our cases, haemostasis and oesophagectomy in the first patient and orthopaedic procedures for the treatment of paralysis, tracheotomy, and drainage to improve sepsis in the second patient were gradually performed before reconstruction. In the first case, graft replacement was considered after TEVAR; however, reconstruction was performed after confirming that the stent infection had subsided during treatment. These results suggest that postoperative complications can be prevented by stabilizing moribund patients through multi-stage surgery. The ability to determine treatment strategies on a case-by-case basis may be a useful aspect of multi-stage surgery.

For oesophageal fistula treatment, an omental flap is recommended to prevent mediastinal infection [13]. We used an omental flap to prevent infectious lesions due to oesophageal fistula. Omental tissue is used in the management of surgical infections because of its high vascularity and neovascularization potential, which helps maintain a stable oxygen supply and an enhanced immune reaction in the infected area [14,15]. In addition, an omental flap can be implanted using various methods; in this study, a pedicled flap was used in the first case and a conjunct flap with a gastric conduit was used in the second case. An important consideration is that a pedicled omental flap obstructs the gastric conduit [9]. Therefore, multi-stage surgery is important to design a suitable method and determine the accurate timing for preliminary delivery of the omental flap to the area of the fistula.

A limitation of this study was the diagnosis with no recurrences. We previously reported that unhealed post-radiotherapy ulcers are associated with a high risk of lethal events such as perforation or penetration within 2 months of observation of the earliest symptom [7]. Therefore, diagnosis of radiotherapy-associated oesophageal fistulas is difficult. The diagnosis of no local recurrence is important when considering dissemination for oesophageal fistula treatment. However, endoscopic biopsy from the irradiated field is considered a precipitating factor for severe bleeding and fistula formation [16]. In our cases, fistula formation and exclusion of recurrence were estimated using CT and MRI, although accurate diagnosis depended on histopathological diagnosis of the resected specimens. The preoperative exclusion of recurrence remains to be addressed.

## 4 Conclusion

We reported two cases involving radiation-induced oesophageal fistula successfully treated by multi-stage surgery without major complications. Multi-stage surgery may be useful for reducing surgical stress in moribund patients.

## 5 Methods

This work has been reported in line with the SCARE 2023 criteria [17].

## Consent

Written informed consent was obtained from the patient for the publication of this case report and the accompanying images. A copy of the written consent form is available for review by the Editor-in-Chief of this journal upon request.

## Ethical approval

This study was performed in accordance with the principles of the Declaration of Helsinki and its later amendments and was approved by the Ethics Committee of Tsukuba University Hospital (No. R1-017).

## Funding

This research did not receive any specific grant from funding agencies in the public, commercial, or not-for-profit sectors.

## CRediT authorship contribution statement

TF and KH drafted the manuscript. JK designed the experiments. All the authors have read and approved the final version of the manuscript.

## Guarantors

The guarantors for this study are Tomoaki Furuta and Katsuji Hisakura.

## Declaration of competing interest

None.
